# Suggested Local Diagnostic Reference Levels for Possible Pediatric X-Ray Optimization in Addis Ababa, Ethiopia

**DOI:** 10.4314/ejhs.v32i1.3S

**Published:** 2022-10

**Authors:** Seife Teferi Dellie

**Affiliations:** 1 Department of Radiology, College of Health Sciences, Addis Ababa University

**Keywords:** X-ray, entrance surface dose, local diagnostic reference levels, Dose Optimization

## Abstract

**Background:**

Early childhood radiation exposure carries an enhanced radiation risk of about two to three times as high as in adults. The objective of this study was to determine local diagnostic reference levels for the most frequent pediatric x-ray examinations in Addis Ababa, Ethiopia.

**Methods:**

A cross-sectional study was conducted on 18 public and private hospitals/clinics in Addis Ababa. A total of 864 pediatric patients, undergoing eight types of routine x-ray examinations with 13 projections were evaluated from December 18/2017 to March 17 /2018. All pediatrics were categorized under four age groups. Anthropometric and radiographic parameters of each patient were recorded .The minimum, maximum, mean and third quartile values of entrance surface dose were analyzed using SPSS version 23. Finally, the suggested local diagnostic reference levels were compared with national and international reference dose values.

**Results:**

Of the 864 pediatric patients, chest (AP/PA), lower and upper extremity (AP/PA) accounted for 501(58%), 110 (13%) and 103 (12%) respectively, accounting to 714(83%) of the total pediatric x-ray examinations. The suggested local diagnostic reference levels of chest X-ray (AP/PA) examinations in mGy were: (0.09,0.13,0.17,0.17) for age group (0-<1,1-<5 ,5-<10, and 10-<15), respectively. Similarly, for same age group (0-<1,1-<5 ,5-<10, and 10-<15) the suggested local diagnostic reference levels of lower and upper extremities in mGy were: (0.06,0.08,0.09,0.09) and (0.04,0.05,0.05,0.06), respectively.

**Conclusion:**

The suggested local diagnostic reference levels were slightly higher than the national and international guidance levels, indicating the need for establishment of diagnostic reference levels in the country.

## Introduction

The clinical value of radiography for the diagnosis of pediatric disease is unquestionable. However, the use is not entirely without risk due to the biological effect of x-rays(1). Inappropriate or unskilled use may result in unnecessary exposures that may increase very high health risk to pediatric patients than adults due to their longer life expectancy during which these effects may manifest (2). It is well known that the risks from ionizing radiation results about two to three fold increase for pediatrics as compared with adult (3,4). Because of smaller body size, children's organs are likely to be within or near the primary beam, so precise collimation more important and more difficult (5). For this reason, improving radiation safety in pediatric imaging has become a global public health issue. This demands policies and actions that recognize and maximize the net benefits that can be obtained, which at the same time minimize potential health risks, achieved by implementing the principles of radiation protection in medicine (6,7).

It is normal for a patient undergoing x-ray examination to expect that the radiation dose received in different hospitals for the same procedure will be within a narrow range. However, wide variations in patient dose for the same type of X-ray examination and for the same examination have been evident from various dose surveys (8–10) which could be attributed to many factors. Among the causes, knowledge and skill of technologists/radiographers are of critical concern, as they are on the first line in radiological practice. In any clinical set-up for any given patient the practice and skill of technologist/radiographer with regard to exposures parameters (like kVp, mAs, FSD, collimation, use of Grids and others) influence both image quality and radiation dose to the patient. A Continuous improvement of radiography techniques and improved image quality with reduced patient dose have been observed through long times. The risk of movement procedure that leads to repeating of the X-ray procedure is also greater with children than adults. This in turn, leads to an increase of the radiation dose to the pediatric patient. For these reasons, it is mandatory to continuously measure pediatric radiation exposure to minimize radiation effects to children. Diagnostic Reference Levels (DRLs) which has been introduced by ICRP in 1996 help to facilitate standardization and optimization within departments and encourage the reduction of dose variations between hospitals (5,11,12). In a research done in the UK, it was noticed that a review of national patient dose enables to reduce patient doses to less than half within 30 years (10). This verifies the benefit of continuing review of reference doss considerations in setting new national diagnostic reference levels (DRLs)(12). To overcome this problem different researchers (12,13) recommends each regulatory body to provide a guidance to assess the situations where the level of patient dose is unusually high and to establish DRLs. Even though pediatric examination and procedures are a special concern as compared with adults, on the contrary few pediatric DRL data are available as compared with adults (14–16). Ethiopia has a legal framework for radiation protection and nuclear protection Proclamation No.1025/2017. The guidance was observed providing some requirements on X-ray units, but does not address the requisites for optimization of protection in medical exposure through the establishment and implementations of DRLs (17). As part of the development of legislation, it was considered important to measure entrance surface dose and provide additional advice to national and local authorities and the clinical communities on the application of DRL as a practical tool to manage radiation dose to the patients so that it commensurate with clinical purpose.

For these reasons the objective of the present research work was to calculate the third quartile (75^th^%) value of entrance surface doses for pediatric patients undergoing common X-ray examinations in eighteen hospitals /clinics Addis Ababa, thereby to suggest the first LDRL, in Addis Ababa, Ethiopia.

## Materials and Methods

**Study design**: A cross-sectional study design was used to study the X-ray unit, the radiation exposure of pediatric patients from December 18/2017 to March 17 /2018 in Addis Ababa. It was designed to define the local diagnostic reference levels for the most common pediatric X-ray examination in Addis Ababa based on 18 hospitals /clinics. The number of hospitals and clinics are limited because of resource availability. Each hospitals and clinics was chosen based on criteria for setting LDRL and on voluntary bass.

**Sample Size and Sampling Technique**: A purposive sampling technique was employed and the sample size was determined based on ICRP recommendations to conduct such study. According to ICRP, such patient dose surveys should include at least 20 standard size patients. Since the study is new in this country, and to increase the precision a minimum of 50 patients with accepted image quality were included from each hospitals/clinics summing up 864 patients. A list of Hospitals/ Clinics was obtained from Ethiopian Ministry of Health and Ethiopian Radiation Protection Authority (ERPA) through personal communication. All hospitals /clinics (public and private) performing pediatric X-ray examination were included. All X-ray machines, radiographers/technologists and all pediatric patients who visited Addis Ababa Hospitals/ clinics were the source Population. While all selected X-ray machines, radiographers and or technologists on duty and pediatric patients were the study population.

**Data collection procedure**: Initially, self-administered questionnaires regarding the X-ray unit was prepared in English and distributed to the radiographers working in the study hospitals/clinics. Completed questionnaires were checked for completeness and consistency and collected from respective institutions.

The tube output (o/p) was measured in a scatter-free geometry, for a peak tube voltage of 80 kVp, 20 current-exposure time (mAs) and a focus-to-detector distance (FFD) of 100 cm, using a calibrated Unfors RaySafe XI dosimeter. While measuring the entrance surface dose in air, relevant anthropometric and exposure parameters (kVp, mAs, FSD) were recorded for each patient undergoing the specified diagnostic procedure. The ESD was calculated in the present work using the following relations.


$$\text{ESD = (O / P)}{\left(\frac{\text{KVP}}{80}\right)}^{2}(\text{mAs)}{\left(\frac{100}{\text{FSD}}\right)}^{2}(\text{BSF)}$$

A value of 1.35 was used in this study for Back Scatter Factor. Eight types of routine x-ray examinations with different projections were evaluated in order to know the higher incidence of projection. These projection were:-Abdomen(AP), Cervical spine(AP), Chest(AP/PA), Lower Extremities(AP/PA), Lumbar spine(AP), Pelvis(AP), Skull(AP/PA+LATERAL) and Upper extremity(AP/PA) x-rays. Besides AP/PA projection, lateral projection was evaluated only for the Skull x-ray, as the ddata on lateral projections of the rest examinations were not dense enough to allow statistical analysis.

As far as effective dose or individual estimation of radiation risk is not a worry, AP and PA projections were not studied in a different way one from another. If effective dose or individual estimation of radiation risk was the concern of this study, then AP position should be studied in a different way from PA, because location of body organs (depth from the surface) differs for both positions and hence different organ dose on AP and PA. To define local diagnostic reference levels (LDRLs) children were categorized into four age groups (0-<1,1-<5 ,5-<10, and 10-<15 in years as given by the EC guidance (18).

**Data Processing and Analysis**: The collected data were inspected for plausibility, and then entered, cleaned and analyzed using SPSS version 24, produced frequency distributions for the variables. Statistical summaries were customized to analyze the mean, maximum and minimum values of the indicated exposure parameters and the mean, maximum, minimum and, third quartile values of calculated ESD were displayed in tables for comparison of national and international values.

**Ethical considerations: -** Ethical considerations were taken into account in order to respect the study group's bill of right. Clear and detailed explanations were given to the family of study population about the objective of the study. Any piece of information was kept confidential by not recording names of respondents.

## Results

In this study, 864 patients from 18 hospitals / clinics undergoing 13 types of projection were recorded during the study period. The frequencies of different X ray projections with four paediatric age groups were documented in table 1. From 864 patients, Chest (AP/PA) accounts 501 (58%) while Upper and lower (AP/PA) extremities accounts 103 (12%) and 110(13 %) respectively. The rest that is, Abdomen AP, skull (AP), skull (PA), skull lateral, pelvic AP, and cervical spine AP accounted for 150 (17%). This is an indication that larger numbers of chest (AP/PA) paediatric X-ray examinations were taken to be the first projections during the research period. While lower and Upper (AP/PA) extremities, were the second and third number of paediatric examinations respectively (table 1).

**Table 1:** Frequencies of paediatric X-ray examination performed between December 18/2017 to March 17 /2018 in Addis Ababa, Ethiopia

AGE GROUP	< 1		1 –< 5		5 –< 10		10 –< 15		Total	

Examination type	Count	%	Count	%	Count	%	Count	%	Count	%
Abdomen AP	16	1.9	4	0.5	6	0.7	3	0.3	29	3%
Cervical spine AP	4	0.5	7	0.8	11	1.3	3	0.3	25	3%
Chest AP/PA	119	13.8	116	13.4	162	18.8	104	12	501	58.%
Lower Ext AP/PA	4	0.5	16	1.9	65	7.5	25	2.9	110	13%
Pelvic AP	2	0.2	4	0.5	12	1.4	7	0.8	25	3%
Skull AP/PA	2	0.2	8	0.9	20	2.3	6	0.7	36	4%
Skull LATERAL	3	0.3	8	0.9	20	2.3	4	0.5	35	4.%
Upper Ext AP/PA	12	1.4	33	3.8	39	4.5	19	2.2	103	12%
TOTAL	162	18.8	196	22.7	335	38.8	171	19.7	864	100%

The intention of this work was to define the local reference levels for the most common standard pediatric X-ray examination and procedures. Hence, even though the contributions of extremities of individual patients were small, they are included in this research together with Chest X-Ray examinations. The mean, range (min, max), of weight, radiographic data, and LDRL of this study along with DRLs published by national and international values for similar age groups and X-Ray projections were documented in table 2. FSD for different paediatric examinations ranges from a minimum of 41 cm to a maximum of 148 cm. The mean kilovolt potential and mill ampere second values were found to be minimum for upper extremity examinations for the less than one year's (48 kVp mean and 1.19mAs mean) and maximum for the paediatric chest examinations in the greater than 10-<15 years (65 kVp mean and 3.89 mAs mean) leading to ESD (third quartile) values of 0.04and 0.17 mGy, respectively.

**Table 2:** The mean, range (min, max ) of weight, radiographic data, and LDRL of the current study along with DRLs published by other author (4,15,19,20) for similar age groups and X-Ray projections

Examination type	This work						National and international DRL	

	Patient age and weight		Radiographic data					
Projection	Age group(Y)	Weight (kg) Mean, (min-max)	kVp mean (min-max)	mAs Mean (min-max)	kVp mean (min-max)	LDRL	UNSCHR(2000)((19),	Paulo G, et al (15)
CXR AP/PA	< 1	6.65(5.3–8.1)	60(40–80)	2.37 (.56–6.25)	104.58, (72–152)	0.09	0.02	0.06
	1 – <5	12.7(10.8–15	56(40–90)	3.32 (1–6.3)	103.3, (46–140)	0.13	0.03	0.07
	5 – <10	19(12.5–24.5)	61(40–80)	3.68,(1–7.88)	115.76, (41–148)	0.17	0.04	0.09
	10 – <15	41.5(26.4–53)	65 (45–90)	3.89(1.4–8.16)	117.94, (53–145)	0.17	0.05	0.09
			**This work**				**Wambani JS etal**(4),	**Rana BS et al** (20)
Lower extremity AP/PA	< 1	8.1(7–8.6)	58 (54–65)	1.4 (1.1–2.0)	98.3, (90–103)	0.06	0.03	0.093
	1 –< 5	10.8(2.7–20)	58,(50–65)	1.98 (1–4)	108, (90–150)	0.08	0.04	0.094
	5 – <10	12.5(5–22)	57 (42–67)	2.15(1.2–3.6)	96.3, (80–110)	0.09	0.05	0.12
	10 – <15	49.5(34–66)	58(42–80)	2.24(0.8–10)	101, (65–150)	0.09	0.05	0.125
Upper extremity AP/PA	< 1	7.1(5.6–8.8)	48 (40–80)	1.19(0.7–1.6)	100, (99–101)	0.04	0.03	0.091
	1 – <5	12.8(8–12)	49(40–62)	1.4 (1–2.12)	102, (72–150)	0.05	0.04	0.098
	5 – <10	24.5(11–26)	53(40–65)	1.57 (1.2–2.5)	99, (75–115)	0.06	0.05	0.141
	10 – <15	26.4(21–33)	55(43–70)	1.92 (1.1–4)	105, (85–150)	0.06	0.05	0.18

## Discussion

In this study, a marked irregularity was observed among operators during their selection of exposure factors. The use of optimal tube potential and tube loading in chest radiography has received a considerable amount of discussion in the radiological literature (4,15,21). As this anatomical structure has excellent subject contrast and to reduce motion blur, it is recommended to employ low mAs and high kVp, 60–65 for neonates, 70–100 for ages 1–5 and 100–120 for ages 5–15. These studies have discouraged the use of tube voltage less than 60 kVp for pediatric patient. The mAs should be 1–2 for age groups 0–5years old and 2–4 for age 5–15years old(18). In this study, the employed kVp does not comply with the mentioned guidance. Mean kVp of 60 for age less than one year and mean kVp of 56 for age >1 – 5 were applied in chest examinations. This suggests that selection of employed exposure factors was random and unstandardized. The appropriate kVp for each specific type of examination is dogmatic, and these values had been optimized over a century of experience. The kVp for each type of conventional radiographic examination should be listed on a technique chart, and this chart should be posted in each radiographic suite(22).

In this study the calculated third quartile ESDs (LDRLs) of chest were found to be greater than the corresponding DRLs of(15) and (19) in all age groups. While lower and upper extremities were found to be higher than (4) and lower than (20)respectively. The main reason for the LDRLs values of chest examinations of (15)studies being lower than the current study is the predominant use of a high kVp , low mAs and high FSD technique with typicall minimum mean values of 77kVp,1.8mAs and 150 cm respectively in all paediatric age groups . This indicates that use of high tube voltage, low tube loading and high FSD techniques reduce Entrance Surface Dose (ESD) by many factors. The LDRLs values obtained for the current paediatric chest X - ray examinations were found to increase with patient age and weight, but in the case of the 5-<10, 10 -<15 years age groups, this relationship was weak. This may be due to the use of low and high (kVp, mAs, FSD) values for ages 5-<10 and 10- <15 years respectively (Table 2). In conclusion, the findings of this study demonstrated that the radiographic technique parameters recorded in this work were inconsistent with international guideline. This can be explained by a long-standing habit of radiographers and or medical radiologic technologists to select their own exposure conditions. All these factors have adverse influences on the outcome of the dose to patients. Such inconsistent use of exposure parameters can be corrected by the use of local diagnostic reference levels. Local diagnostic reference levels can facilitate standardization and optimization within departments and encourage the reduction of dose variations between hospitals (5).

In conclusion, the author of this manuscript concludes that, the values of local diagnostic reference levels (LDRLs) presented in this work are suitable to be adopted for the paediatric diagnostic X-ray examinations in Addis Ababa, Ethiopia. It can be used as a baseline upon which future dose measurements can be compared. Furthermore, similar type of large-scale survey should be undertaken to establish national DRLs in the case of paediatric X-ray examinations.
